# Supplementary material for: Performance of Blood-Based Biomarkers for Human Circadian Pacemaker Phase: Training Sets Matter As Much As Feature-Selection Methods
Source: J Biol Rhythms. 2025 Aug 31;40(6):506–27. doi: 10.1177/07487304251358950 (PMC12572356; doi:10.1177/07487304251358950)
Supplement: sj-pdf-1-jbr-10.1177_07487304251358950 – Supplemental material for Performance of Blood-Based Biomarkers for Human Circadian Pacemaker Phase: Training Sets Matter As Much As Feature-Selection Methods [file sj-pdf-1-jbr-10.1177_07487304251358950.pdf]

Supplemental Material

A

	Condition	Study	Number of samples (*N*)
IP	Sleeping in phase	Forced desynchrony	156
OP	Sleeping out of phase	Forced desynchrony	146
SS	Sufficient sleep	Sleep restriction	242
IS	Insufficient sleep	Sleep restriction	238
BD	Baseline 1	Bed rest	107
BD	Baseline 2	Bed rest	107
HDT	Bed rest 1	Bed rest	112
HDT	Bed rest 2	Bed rest	108
HDT	Bed rest 3	Bed rest	102
R	Recovery	Bed rest	101
			Total = 1,419

B

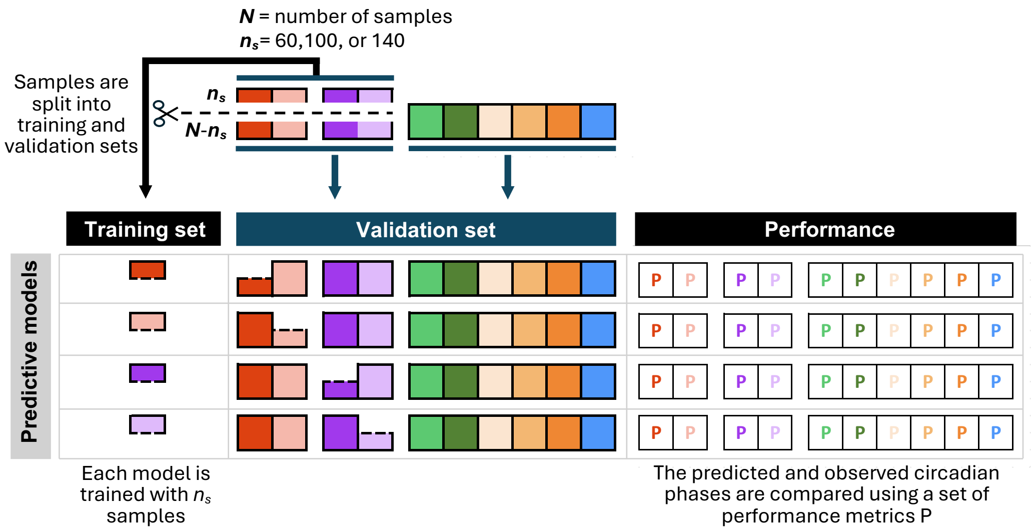

Supplemental figure 1. Methodological approach. A Description of experimental conditions and number of samples. **B** Diagram of methodological approach. Training sets were created by randomly selecting n_s samples from the total *N* samples of condition *X*. We used two selection strategies: one in which samples were randomly selected, and another in which samples were also randomly selected, but with no overlap between participants in the training and validation sets. The validation set included the remaining (N-n_s) samples from condition *X*, along with all samples from other conditions. A predictive model was developed on each training set and used to predict circadian phase in the validation set. The predicted and observed circadian phase were compared, and a set of performance metrics (P) were calculated independently for each experimental condition in the validation set.

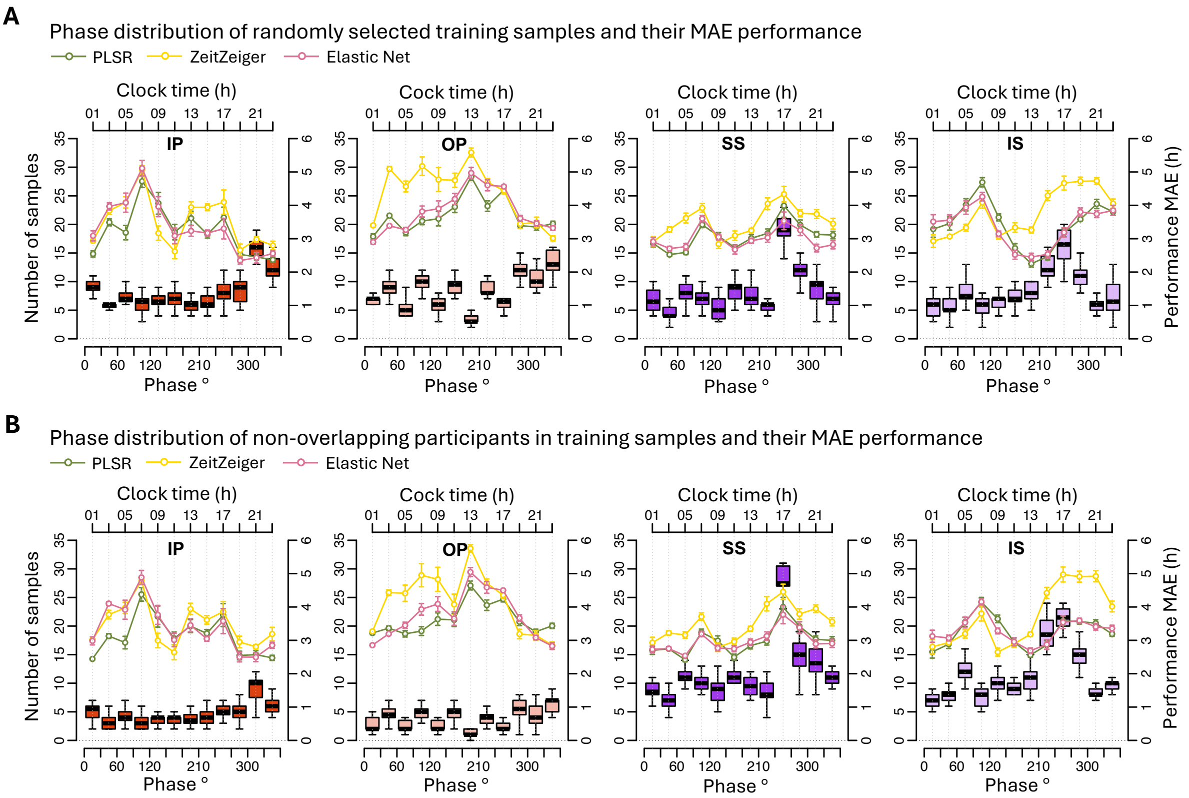

C

Correlation between mean MAE values in Panels A and B

	IP	OP	SS	IS
**PLSR**	0.97	0.8	0.94	0.96
**ZZ**	0.97	0.98	0.96	0.94
**EN**	0.98	0.83	0.94	0.81

Supplemental Figure 2. Effect of training sample selection strategy on phase distribution and prediction performance across the circadian cycle. Panel A shows results from training sets composed of randomly selected samples (with possible participant overlap), while **panel B** shows results from training sets constructed using non-overlapping participants. For each of the four training conditions, the number of training samples (boxplots, left y-axis) and prediction performance (mean absolute error [MAE], line plots, right y-axis) are shown as a function of circadian phase (°, bottom axis) and clock time (h, top axis). Boxplots represent the distribution of sample counts across 30° phase bins from 20 random draws of 100 samples. MAE values for three prediction methods - PLSR (green), ZeitZeiger (yellow), and Elastic Net (pink) - reflect the average MAE across the four validation sets, with error bars indicating standard deviation. Panel **C** presents the Pearson correlation between the MAE values in panels A and B (n=12 per cell). All Benjamini–Hochberg (BH)-corrected correlation p-values were < 0.005. In all panels, columns correspond to the conditions used to derive the training sets. IP = sleeping in phase during the night, OP = sleeping out of phase during the day, SS = constant wake after one week of sufficient sleep, IS = constant wake after one week of insufficient sleep.

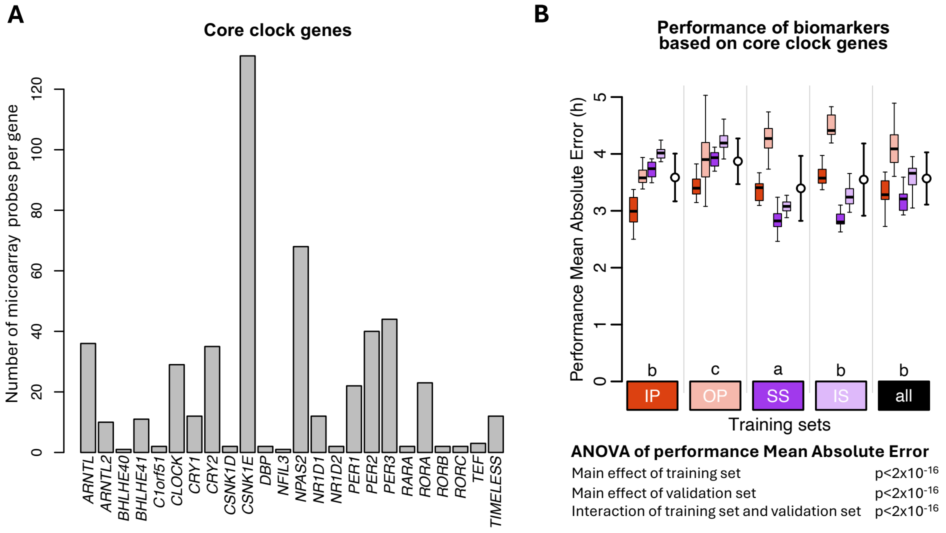

C

Performance Mean Average Error
mean (standard deviation)

IP	OP	SS	IS	all
3.6 (0.42)	3.9 (0.4)	3.4 (0.57)	3.5 (0.63)	3.6 (0.46)

D

Performance Mean Average Error of training set
mean (standard deviation)

IP	OP	SS	IS	all
1.5 (0.087)	1.7 (0.11)	1.5 (0.09)	1.6 (0.11)	1.7 (0.13)

ANOVA of performance Mean Averaged Error
Main effect of training set p=3.46x10^{-11}

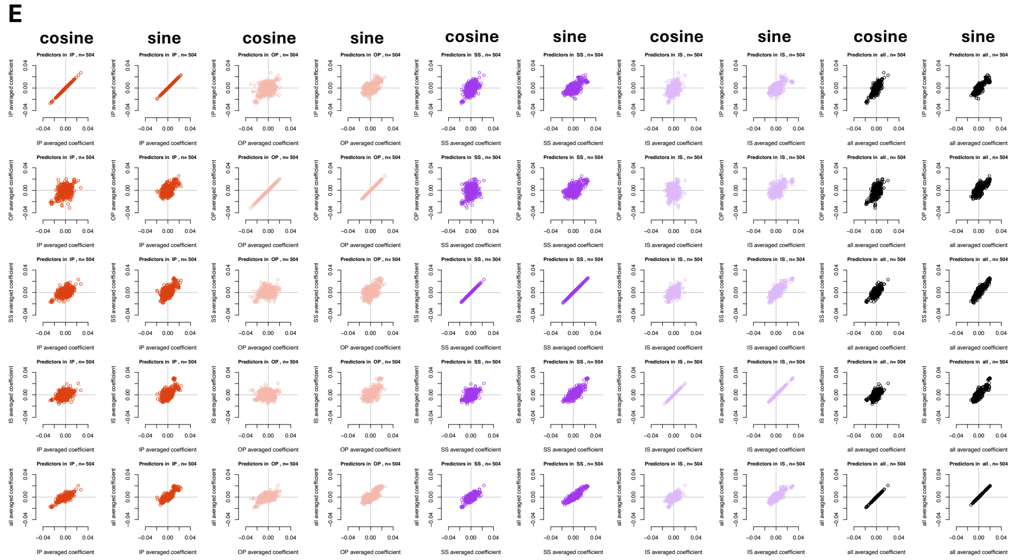

Supplemental figure 3. PLSR based on a fix set of clock genes. A 24 core clock genes were mapped to 504 microarray probes and used as predictors in the generation of

PLSR biomarkers. **B** Performance results obtained for PLSR. Twenty training sets of 100 samples were generated for each experimental condition. Each coloured box plot represents the distribution of 20 mean absolute error (MAE) values for each condition in the validation set, with outliers omitted for clarity. White open circles with error bars indicate the mean and standard deviation of all 80 validation values per training set (20 sets x 4 conditions). The two-way ANOVA for MAE showed significant effects of both the training set and validation set as well as their interaction (all $p < 2\times10^{-16}$). The Compact Letter Display (CLD) summarizes the results of pairwise comparisons between different training sets, as determined by Tukey's test conducted on the two-way ANOVA of training and validation sets. **C** Mean and standard deviation of 80 MAE values per training condition (20 training sets x 4 validation conditions) shown in panel **A**. **D** Mean and standard deviation of 20 MAE values per training condition (20 training sets per condition), where the same samples are used for both training and validation. The ANOVA for MAE of the training set showed significant effect of training set ($p=3.46\times10^{-11}$). In all panels, training sets are depicted as coloured rectangles on the horizontal axis. **E** Averaged regression coefficients (across the 20 runs) of the 504 predictor transcripts in the models generated in panel **A**. IP = sleeping in phase during the night, OP = sleeping out of phase during the day, SS = constant wake after one week of sufficient sleep, IS = constant wake after one week of insufficient sleep. The condition "all" includes an equal number of samples from each of the four experimental conditions. IP = sleeping in phase during the night, OP = sleeping out of phase during the day, SS = constant wake after one week of sufficient sleep, IS = constant wake after one week of insufficient sleep.

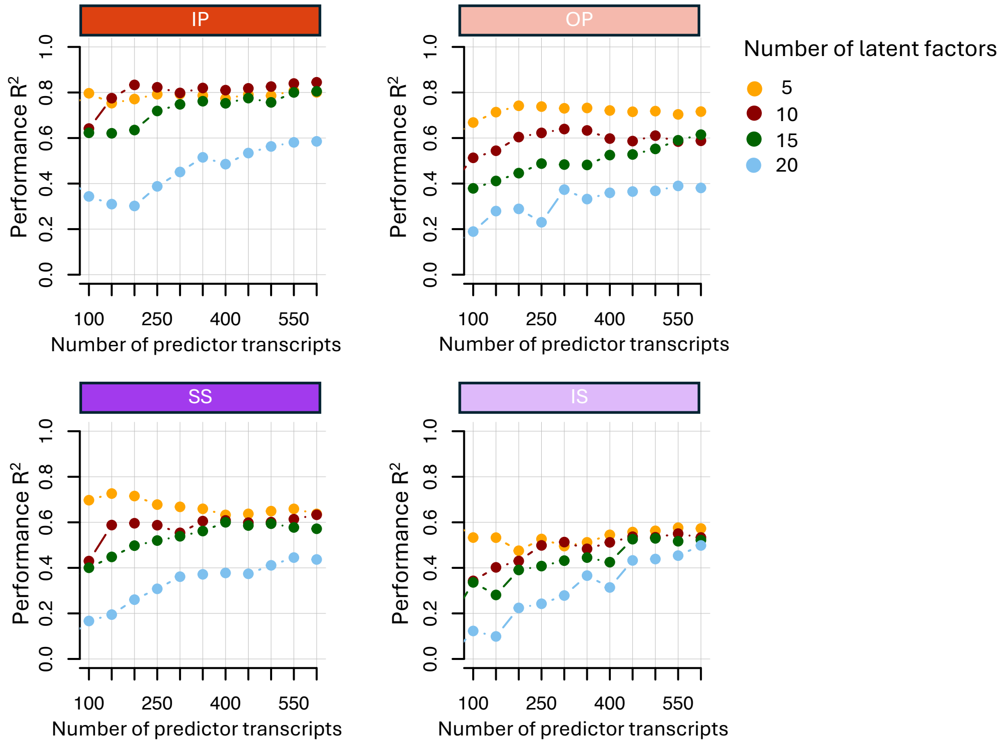

Supplemental Figure 4. Selection of number of predictor transcripts and latent factors for PLSR models. Leave-one-participant-out cross-validation performance R^2 applied to training set when using different combinations of number of predictor

transcripts (horizontal axis) and latent factors (coloured circles). Each panal corresponds to a different training set condition. All training sets were restricted to 140 samples.

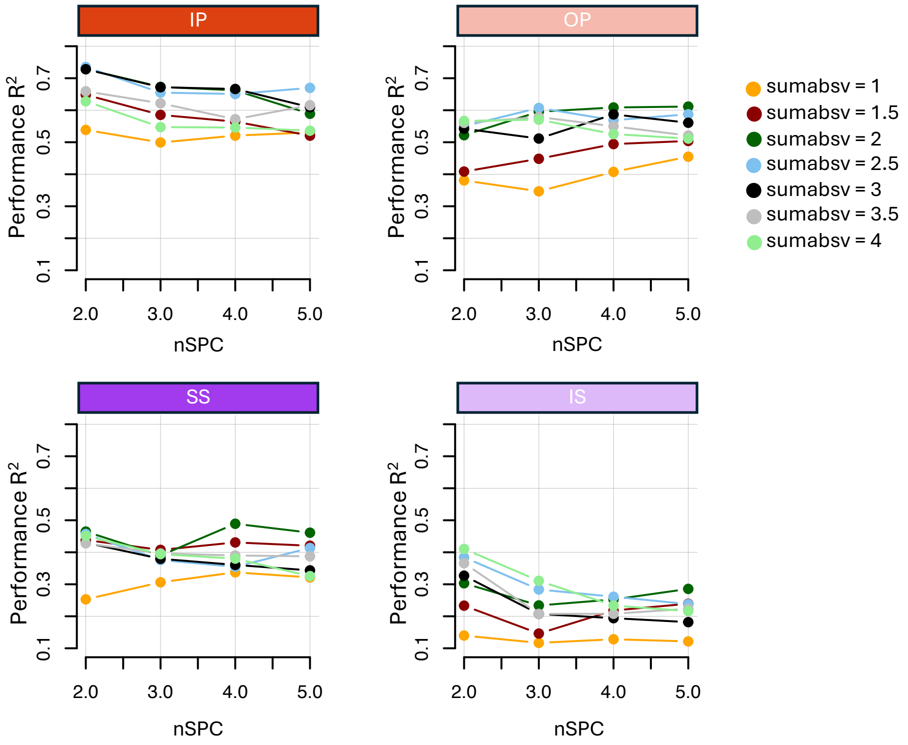

Supplemental Figure 5. Selection of nSPC and sumabsv values for ZZ models. Leave-one-participant-out cross-validation performance R2 applied to training set when using different combinations of nSPC (horizontal axis) and sumabsv (coloured circles) values. Each penal corresponds to a different training set condition. All training sets were restricted to 140 samples.

A

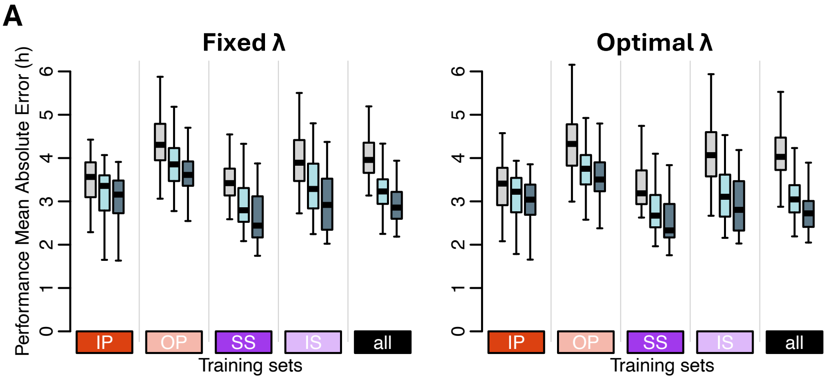

B

Performance Mean Absolute Error mean (standard deviation)

	n_s	IP	OP	SS	IS	all
Fixed λ	60	3.5 (0.6)	4.4 (0.6)	3.5 (0.5)	4.0 (0.6)	4.0 (0.5)
	100	3.2 (0.6)	3.9 (0.5)	3.0 (0.6)	3.4 (0.6)	3.3 (0.4)
	140	3.0 (0.7)	3.5 (0.7)	2.6 (0.6)	3.0 (0.7)	2.9 (0.5)
Optimal λ	60	3.4 (0.6)	4.4 (0.7)	3.4 (0.7)	4.1 (0.7)	4.1 (0.6)
	100	3.1 (0.6)	3.7 (0.6)	2.8 (0.5)	3.2 (0.6)	3.1 (0.5)
	140	2.9 (0.7)	3.4 (0.7)	2.5 (0.6)	2.9 (0.7)	2.8 (0.5)

C

Number of overlapping predictors

Transcripts appearing in at least 10 out of 20 models generated per training condition

	n_s	IP	OP	SS	IS	all
Fixed λ	60	9	6	6	4	2
	100	39	33	14	15	5
	140	147	209	46	42	11
Optimal λ	60	6	4	2	2	1
	100	30	18	15	11	5
	140	104	67	35	31	9

Supplemental Figure 6. Effect of lambda selection (fixed vs. optimised) on EN model performance. A Performance results obtained for two lambda selection strategies: Fixed (left) and Optimal (right). Each box plot represents the distribution of 20 mean absolute error (MAE) values for each condition of the validation set, totalling 80 values (20 sets x 4 conditions), with outliers omitted for clarity. **B** Mean and standard deviation of 80 MAE values per training condition (20 training sets x 4 validation conditions) shown in panel **A**. **C** Number of overlapping predictors, defined as transcripts appearing in at least 10 out of the 20 models generated per training set. In each panel, results were obtained for n_s values of 60 (gray box plots), 100 (light blue box plots) and 140 (dark blue box plots), and training sets are represented by coloured rectangles on the horizontal axis. The training condition “all” includes an equal number of samples from each of the four experimental conditions. IP = sleeping in phase during the night, OP = sleeping out of phase during the day, SS = constant wake after one week of sufficient sleep, IS = constant wake after one week of insufficient sleep.

Effect of number of samples in the training set on biomarker performance

Performance Mean Error mean (standard deviation)
rows = method _ number of samples in training set
columns = training set

	IP	OP	SS	IS	all
PLSR_60	0.35 (1.2)	2.2 (1.5)	-0.48 (1.1)	-1.4 (1.3)	-0.17 (1.3)
PLSR_100	0.3 (1.2)	1.7 (1.1)	-0.43 (0.95)	-1.5 (1)	0.096 (1.1)
PLSR_140	0.21 (1.2)	1.5 (1.4)	-0.33 (0.92)	-1.3 (0.93)	-0.077 (0.96)
ZZ_60	0.41 (1.3)	2.2 (1.6)	-0.34 (1.1)	-1.3 (1.2)	-0.061 (1.3)
ZZ_100	0.47 (1.2)	2 (1.3)	-0.55 (1)	-1.3 (1)	-0.034 (1)
ZZ_140	0.67 (1.3)	1.8 (1.8)	-0.5 (1.1)	-1.2 (0.97)	-0.084 (1)
EN_60	0.35 (1.2)	2.1 (1.4)	-0.47 (1)	-1.5 (1.3)	-0.15 (1.3)
EN_100	0.26 (1.2)	1.8 (1.2)	-0.49 (0.93)	-1.4 (0.96)	-0.097 (1)
EN_140	0.31 (1.2)	1.4 (1.4)	-0.46 (0.94)	-1.3 (0.89)	-0.17 (0.96)

Performance Mean Error of training set mean (standard deviation)
rows = method _ number of samples in training set
columns = training set

	IP	OP	SS	IS	all
PLSR_60	0.015 (0.022)	0.0039 (0.04)	0.0056 (0.027)	-0.0025 (0.034)	-0.012 (0.023)
PLSR_100	0.053 (0.034)	0.011 (0.047)	0.014 (0.039)	-0.0058 (0.038)	0.015 (0.047)
PLSR_140	0.044 (0.035)	0.018 (0.058)	0.0065 (0.041)	-0.025 (0.049)	0.0066 (0.061)
ZZ_60	0.039 (0.16)	-0.059 (0.21)	0.045 (0.22)	-0.042 (0.2)	0.013 (0.26)
ZZ_100	-0.012 (0.12)	-0.066 (0.18)	0.069 (0.16)	-0.051 (0.26)	-0.0094 (0.2)
ZZ_140	0.026 (0.14)	-0.038 (0.1)	0.087 (0.13)	-0.0029 (0.19)	-0.038 (0.18)
EN_60	0.00018 (0.00061)	0.00021 (0.00043)	-0.00025 (0.00064)	-0.00019 (0.00054)	-9.5e-05 (0.00066)
EN_100	0.00084 (0.00065)	0.00031 (0.00048)	-0.00038 (0.0011)	-0.00086 (0.00097)	2.2e-06 (0.00075)
EN_140	0.0018 (0.00071)	0.0017 (0.00058)	4.1e-05 (0.0015)	-0.0018 (0.0014)	-9.6e-05 (0.0013)

Effect of training set and validation set on biomarker performance

Performance Mean Error mean (standard deviation)
rows = method
columns = training set

	IP	OP	SS	IS	all
PLSR	0.21 (1.2)	1.5 (1.4)	-0.33 (0.92)	-1.3 (0.93)	-0.077 (0.96)
ZZ	0.67 (1.3)	1.8 (1.8)	-0.5 (1.1)	-1.2 (0.97)	-0.084 (1)
EN	0.31 (1.2)	1.4 (1.4)	-0.46 (0.94)	-1.3 (0.89)	-0.17 (0.96)

Performance Mean Error of training set mean (standard deviation)
rows = method
columns = training set

	IP	OP	SS	IS	all
PLSR	0.044 (0.035)	0.018 (0.058)	0.0065 (0.041)	-0.025 (0.049)	0.0066 (0.061)
ZZ	0.026 (0.14)	-0.038 (0.1)	0.087 (0.13)	-0.0029 (0.19)	-0.038 (0.18)
EN	0.0018 (0.00071)	0.0017 (0.00058)	4.1e-05 (0.0015)	-0.0018 (0.0014)	-9.6e-05 (0.0013)

Performance of biomarkers on an independent data set collected in a bed rest protocol.

Performance Mean Error mean (standard deviation)
rows = method
columns = training set

	IP	OP	SS	IS	all
PLSR	0.2 (0.43)	2 (0.66)	-0.53 (0.46)	-1.2 (0.46)	-0.057 (0.75)
ZZ	0.46 (0.45)	2.1 (0.48)	0.063 (0.44)	-0.96 (0.5)	0.2 (0.59)
EN	0.79 (0.42)	2 (0.68)	-0.24 (0.46)	-1.2 (0.55)	0.08 (0.71)

Effect of Training and Validation Sets on Biomarker Performance When Using Core Clock Genes for Training

Performance Mean Error mean (standard deviation)
rows = method
columns = training set

	IP	OP	SS	IS	all
PLSR	0.38 (0.99)	1.8 (1.2)	-0.31 (0.9)	-1.3 (1.1)	-0.047 (1.2)

Performance Mean Error of training set mean (standard deviation)
rows = method
columns = training set

	IP	OP	SS	IS	all
PLSR	0.059 (0.061)	0.059 (0.078)	0.034 (0.08)	0.04 (0.056)	0.014 (0.1)

Table S1. Performance results based on Mean Error

Effect of number of samples in the training set on biomarker performance

Performance R2 mean (standard deviation)
rows = method _ number of samples in training set
columns = training set

	IP	OP	SS	IS	all
PLSR_60	0.42 (0.12)	0.18 (0.17)	0.37 (0.11)	0.29 (0.11)	0.29 (0.1)
PLSR_100	0.55 (0.13)	0.37 (0.14)	0.54 (0.12)	0.46 (0.12)	0.45 (0.1)
PLSR_140	0.59 (0.12)	0.37 (0.41)	0.63 (0.13)	0.57 (0.13)	0.57 (0.099)
ZZ_60	0.37 (0.1)	0.14 (0.14)	0.35 (0.11)	0.25 (0.12)	0.26 (0.092)
ZZ_100	0.44 (0.11)	0.22 (0.14)	0.42 (0.082)	0.34 (0.11)	0.37 (0.086)
ZZ_140	0.45 (0.14)	0.19 (0.46)	0.43 (0.074)	0.37 (0.1)	0.41 (0.1)
EN_60	0.45 (0.13)	0.21 (0.17)	0.43 (0.11)	0.3 (0.13)	0.3 (0.1)
EN_100	0.54 (0.14)	0.33 (0.15)	0.57 (0.11)	0.47 (0.13)	0.48 (0.097)
EN_140	0.59 (0.13)	0.36 (0.33)	0.66 (0.12)	0.56 (0.14)	0.56 (0.1)

Performance R2 of training set mean (standard deviation)
rows = method _ number of samples in training set
columns = training set

	IP	OP	SS	IS	all
PLSR_60	0.98 (0.0066)	0.97 (0.0087)	0.98 (0.0084)	0.97 (0.0075)	0.97 (0.011)
PLSR_100	0.94 (0.018)	0.94 (0.014)	0.94 (0.016)	0.94 (0.017)	0.93 (0.025)
PLSR_140	0.92 (0.012)	0.91 (0.0091)	0.92 (0.015)	0.91 (0.015)	0.9 (0.024)
ZZ_60	0.84 (0.077)	0.79 (0.06)	0.76 (0.085)	0.73 (0.12)	0.79 (0.072)
ZZ_100	0.78 (0.052)	0.72 (0.061)	0.65 (0.072)	0.62 (0.075)	0.67 (0.07)
ZZ_140	0.75 (0.054)	0.71 (0.034)	0.6 (0.05)	0.58 (0.07)	0.63 (0.071)
EN_60	1 (0.00013)	1 (8.3e-05)	1 (9.4e-05)	1 (0.00014)	1 (9.2e-05)
EN_100	1 (0.00019)	1 (0.00012)	1 (0.00012)	1 (0.00019)	1 (0.00012)
EN_140	1 (0.00013)	1 (6.9e-05)	1 (0.00016)	1 (0.00025)	1 (2e-04)

Effect of training set and validation set on biomarker performance

Performance R2 mean (standard deviation)
rows = method
columns = training set

	IP	OP	SS	IS	all
PLSR	0.59 (0.12)	0.37 (0.41)	0.63 (0.13)	0.57 (0.13)	0.57 (0.099)
ZZ	0.45 (0.14)	0.19 (0.46)	0.43 (0.074)	0.37 (0.1)	0.41 (0.1)
EN	0.59 (0.13)	0.36 (0.33)	0.66 (0.12)	0.56 (0.14)	0.56 (0.1)

Performance R2 of training set mean (standard deviation)
rows = method
columns = training set

	IP	OP	SS	IS	all
PLSR	0.92 (0.012)	0.91 (0.0091)	0.92 (0.015)	0.91 (0.015)	0.9 (0.024)
ZZ	0.75 (0.054)	0.71 (0.034)	0.6 (0.05)	0.58 (0.07)	0.63 (0.071)
EN	1 (0.00013)	1 (6.9e-05)	1 (0.00016)	1 (0.00025)	1 (2e-04)

Performance of biomarkers on an independent data set collected in a bed rest protocol.

Performance R2 mean (standard deviation)
rows = method
columns = training set

	IP	OP	SS	IS	all
PLSR	0.69 (0.053)	0.37 (0.13)	0.61 (0.093)	0.57 (0.13)	0.48 (0.13)
ZZ	0.57 (0.092)	0.39 (0.077)	0.46 (0.094)	0.47 (0.11)	0.49 (0.11)
EN	0.55 (0.084)	0.29 (0.12)	0.56 (0.094)	0.41 (0.14)	0.43 (0.12)

Effect of Training and Validation Sets on Biomarker Performance When Using Core Clock Genes for Training

Performance R2 mean (standard deviation)
rows = method
columns = training set

	IP	OP	SS	IS	all
PLSR	0.41 (0.11)	0.31 (0.12)	0.48 (0.11)	0.44 (0.13)	0.41 (0.096)

Performance R2 of training set mean (standard deviation)
rows = method
columns = training set

	IP	OP	SS	IS	all
PLSR	0.9 (0.019)	0.85 (0.028)	0.89 (0.022)	0.84 (0.031)	0.86 (0.03)

Table S2. Performance results based on R^2

Performance Mean Absolute Error mean (standard deviation)

rows = validation set
columns = training set

PLSR

	IP	OP	SS	IS	all
BDC1	2.3 (0.12)	3.9 (0.41)	2.9 (0.35)	3.2 (0.41)	3.4 (0.45)
BDC2	2.5 (0.24)	4.3 (0.39)	2.9 (0.31)	3.1 (0.57)	3.3 (0.52)
HDT1	2.7 (0.21)	3.7 (0.37)	3.1 (0.38)	3.2 (0.46)	3.4 (0.46)
HDT2	2.6 (0.21)	3.6 (0.39)	2.8 (0.32)	3 (0.52)	3.3 (0.49)
HDT3	2.7 (0.19)	3.6 (0.43)	2.6 (0.36)	3 (0.57)	3.5 (0.48)
R	2.8 (0.29)	4.3 (0.4)	2.9 (0.4)	3 (0.49)	3.6 (0.42)

ZeitZeiger

	IP	OP	SS	IS	all
BDC1	3.1 (0.47)	3.9 (0.19)	4 (0.28)	3.9 (0.44)	3.6 (0.51)
BDC2	2.9 (0.37)	4 (0.21)	3.3 (0.14)	3.5 (0.39)	3.3 (0.44)
HDT1	3.1 (0.38)	3.7 (0.23)	3.2 (0.23)	3.4 (0.52)	3.4 (0.37)
HDT2	3.1 (0.35)	4.2 (0.16)	3.9 (0.23)	3.8 (0.36)	3.6 (0.37)
HDT3	3.3 (0.29)	3.8 (0.32)	3.5 (0.26)	3.5 (0.33)	3.3 (0.42)
R	3.4 (0.26)	4.1 (0.28)	3.2 (0.21)	3.3 (0.6)	3.5 (0.51)

Elastic Net

	IP	OP	SS	IS	all
BDC1	2.8 (0.29)	4.2 (0.33)	3.4 (0.32)	3.8 (0.41)	3.8 (0.48)
BDC2	3 (0.31)	4.7 (0.38)	3.1 (0.44)	3.6 (0.65)	3.7 (0.53)
HDT1	2.8 (0.26)	3.8 (0.3)	3.3 (0.46)	3.8 (0.49)	3.7 (0.53)
HDT2	3.1 (0.44)	4.2 (0.44)	3.2 (0.46)	3.7 (0.54)	3.6 (0.48)
HDT3	3.1 (0.26)	4 (0.29)	3 (0.43)	3.8 (0.57)	3.7 (0.46)
R	3.4 (0.29)	4.5 (0.28)	3.3 (0.5)	3.9 (0.48)	4 (0.39)

Table S3. Performance based on an independent dataset

Study	Prediction method	Training sleep/wake condition	Phase marker	Training set size (samples/participants); biological sample	Validation	Predictor error	Predictors/annotation
Hughey 2017	ZeitZeiger; spline fitting, maximum likelihood	Constant routine; FD sleeping in phase; normal sleep/wake in lab	Sunrise; average DLMO	498/60; whole blood	Ten-fold cross validation; LOOCV	Median absolute error 2.1h (1 sample)	15 genes; 2 clock genes (*PER1* , *NR1D2*); 6 glucocorticoid signalling genes (*DDIT4* , *FKBP5* , *CLEC4E* , *DAAM2* , *PER1* , *NR1D2*)
Wu et al., 2018	ZeitZeiger; spline fitting, maximum likelihood	Normal sleep/wake in lab	Saliva melatonin/cortisol	219 (no time)/219, 43 (timed)/20; skin epidermis	Independent cohort; 9 participants not included in training set	Mean absolute error 2.95h (1 sample)	29 genes; 6 clock genes (*ARNTL* , *NR1D1* , *NR1D2* , *PER1* , *PER3* , *CIART*); 4 glucocorticoid signalling genes (*FKBP5* , *NR1D2* , *PER1* , *ZBTB16*)
Wittenbrink et al., 2018	ZeitZeiger; spline fitting, maximum likelihood	Constant routine	Saliva DLMO	136/10; isolated monocytes	LOOCV; independent cohort validation in extreme chronotypes (n=28)	Median absolute error 0.69h morning and 0.80h afternoon samples (1 sample, 12 gene); 0.74h morning + afternoon samples (2 samples, 13 gene)	2 sample, 13 gene model; *PER2* , *PER3* , *NR1D1* , *NR1D2* , *CRY1* , *KLF9* , *CRISPLD2* , *LGALS3* , *ELMO2* , *FKBP4*, *TSPAN4*, *CX3CR1*, *CPED1*
Laing et al., 2018	Partial least squares regression	Constant routine after one week of sufficient and insufficient sleep; FD sleeping in and out of phase	Plasma DLMO	329/26; whole blood	349 samples from 27 participants not included in training set	1 sample, mean error -18mins, SD of error 3:17 h:mins; mean absolute error 2:28 h:mins, SD of absolute error 2:12 h:mins; 2 samples, mean error -16mins, SD of error 2:39 h:mins	1 sample, 100 transcripts targeting 80 genes; 2 sample, 100 transcripts targeting 76 genes; both include clock genes (*PER1* , *PER2* , *NR1D2*) and glucocorticoid signalling genes (*PER1* , *NR1D2* , *DDIT4* , *FKBP5* , *ZBTB16* , *FLT3*)
Braun et al., 2018; Braun et al., 2019	TimeSignature; sine fitting, elastic net regression	Constant routine after normal or restricted sleep	Time of day; time since DLMO25%	240/12; whole blood	Ten-fold cross validation; independent cohorts for sleeping in/out of phase, sleep deprivation, normal sleep/wake in lab	Median absolute error 1.28h (time of day); 1.12h (time since DLMO25%) (1 sample plus 1 within-subject normalisation sample)	41 genes; 3 clock genes (*PER1* , *NR1D1* , *NR1D2*); 4 glucocorticoid signalling genes (*NR1D2* , *PER1* , *DDIT4* , *FKBP4*)
Lee et al., 2020	Endogenous Circadian rhythm Estimation Model (eCEM); Cosine fitting, least squares regression	Normal sleep/wake at home	Time of day	179/18; hair follicles	LOOCV	Mean absolute error 3.436h (1 sample)	10 predefined clock genes (*PER1* , *PER2* , *PER3* , *CLOCK* , *ARNTL* , *CRY1* , *CRY2* , *NPAS2* , *NR1D1* , *NR1D2*)

Table S4. Comparison of studies that have used different approaches to develop transcriptomic predictors of circadian timing.
